# Lateral Transport of Organic and Inorganic Solutes

**DOI:** 10.3390/plants8010020

**Published:** 2019-01-15

**Authors:** Emilie Aubry, Sylvie Dinant, Françoise Vilaine, Catherine Bellini, Rozenn Le Hir

**Affiliations:** 1 Institut Jean-Pierre Bourgin, INRA, AgroParisTech, CNRS, Université Paris-Saclay, 78000 Versailles, France; emilie.aubry@inra.fr (E.A.); sylvie.dinant@inra.fr (S.D.); francoise.vilaine@inra.fr (F.V.); catherine.bellini@inra.fr (C.B.); 2 Umeå Plant Science Centre, Department of Plant Physiology, Umeå University, 90183 Umeå, Sweden

**Keywords:** phloem, xylem, lateral transport, organic solutes, inorganic solutes

## Abstract

Organic (e.g., sugars and amino acids) and inorganic (e.g., K^+^, Na^+^, PO_4_^2−^, and SO_4_^2−^) solutes are transported long-distance throughout plants. Lateral movement of these compounds between the xylem and the phloem, and vice versa, has also been reported in several plant species since the 1930s, and is believed to be important in the overall resource allocation. Studies of *Arabidopsis thaliana* have provided us with a better knowledge of the anatomical framework in which the lateral transport takes place, and have highlighted the role of specialized vascular and perivascular cells as an interface for solute exchanges. Important breakthroughs have also been made, mainly in Arabidopsis, in identifying some of the proteins involved in the cell-to-cell translocation of solutes, most notably a range of plasma membrane transporters that act in different cell types. Finally, in the future, state-of-art imaging techniques should help to better characterize the lateral transport of these compounds on a cellular level. This review brings the lateral transport of sugars and inorganic solutes back into focus and highlights its importance in terms of our overall understanding of plant resource allocation.

## 1. Introduction

In plants, organic (e.g., organic acids, sugars, amino acids) and inorganic (e.g., K^+^, Na^+^, NH_4_^+^, NO^3−^, SO_4_^2−^, PO_4_^2−^, Ca^2+^) solutes are dissolved in a water-based mixture (i.e., phloem and xylem sap) that is distributed throughout the plant. Understanding how these nutrients are transported between organs and how this transport is coordinated is of major interest for the long-term goal of improving resource allocation in plants and *in fine* plant biomass production. The solute movement between and within the different organs or tissues of a plant depends on the plant’s developmental stage and on the environmental conditions that the plant is facing. Nevertheless, depending on the type of compound, different transport systems act in a complementary way to allow the solutes to reach their targeted tissue and cell (i.e., long-distance versus short-distance transport, apoplasmic versus symplasmic pathway).

Over the long-distance, the transport of water and nutrients is achieved by the plant’s vascular system, which is composed of phloem and xylem tissues [1]. In addition, several compounds, including sugars [2], amino acids [2], minerals [3], ions [4], water [5], miRNA [6], transcription factors [6,7,8,9], hormones [10,11], secondary metabolites [12,13], and more complex molecules like monolignols [14,15], have been shown to be transported inside the different vascular cell types and between the vascular and the perivascular tissues (i.e., the endodermis, the pericycle, and vascular parenchyma cells). More specifically, when addressing the cell-to-cell movement of solutes (e.g., sugars, amino acids, and ions), authors refer equally to “solute exchange” [16], “radial solute exchange”, “radial transport” [2,5], or “lateral transport” [17]. Moreover, this cell-to-cell movement has in some cases been associated with transport between specific tissues in a preferential direction, such as “xylem-to-phloem transfer” or “phloem-to-xylem transfer” [2,18,19].

During the 1960s and 1970s, extensive literature addressing lateral transport in various species was published, and was last reviewed by van Bel in 1990 [2]. These studies unequivocally stress the importance of this process in overall plant resource allocation. Since then, the use of the model plant *Arabidopsis thaliana* has allowed significant progress to be made in the identification of the molecular actors involved in such processes, even if direct experimental proof of lateral transport in this species is scarce (Table 1). After presenting experimental evidence for the existence of lateral transport in various plant species, this review will detail the anatomical framework in which solute exchanges take place, as well as the molecular actors involved in the process, mostly identified in *Arabidopsis thaliana*. Of the various organic and inorganic solutes for which lateral transport has been shown (Table 1), this review will not address (or will only mention briefly) xylem-to-phloem transport of calcium, magnesium, sulfate, and nitrogen, because this has been reviewed elsewhere [20,21,22,23,24]. Instead, the review will focus on the transport of photosynthates and on the transport of potassium, sodium, and phosphate as inorganic ions.

## 2. Evidence for the Existence of Lateral Transport in Vascular Plants

Stout and Hoagland first reported the experimental existence of lateral transport at the end of the 1930s. Radioactive isotopes of potassium, sodium, and phosphorus were used to show that these ions were transported from xylem to phloem in geranium (*Pelargonium zonale*) and in willow (*Salix lasiandra*) [25]. Later, during the 1960s and 1970s, numerous articles were published that addressed the lateral transport of organic and inorganic solutes in both herbaceous and woody species (Table 1 and references therein). Most of these studies also used radio-labelled sugars, amino acids, or ions to support their conclusions. Interestingly, not all inorganic ions are equal with regard to lateral transport. According to Pate (1975), a solute should fulfil several physiological criteria in order to move freely within the plant, such as a low degree of involvement in organic linkage, rapid cycling through the leaves, and a high concentration in the vascular tissues [16]. Among the different inorganic ions, potassium (K^+^), phosphate (PO_4_^2−^), and sulfate (SO_4_^2−^) come closest to meeting all of these criteria. Sodium (Na^+^) is also transferred from xylem to phloem, but to a lesser extent than K^+^, while calcium ions fail to cross from xylem to phloem, even if perfused at a high concentration [26,27]. Regardless of the plant species, amino acids mainly move from xylem to phloem, while sugars move in both phloem-to-xylem and xylem-to-phloem directions (Table 1). For instance, Van Bel (1984) demonstrated in tomato plants (*Lycopersicum esculentum*) that the nutritional demands of young leaves were almost completely met by xylem-to-phloem transport of amino acids in the tomato stem [28]. In the legume species, *Lupinus albus*, it was shown that vegetative apices received 73% of their N and 14% of their C through the xylem [29], while in soybean (*Glycine max*), 6% of C and 51% of N enters the fruit via xylem-to-phloem transfer in the stem [30]. Phloem-to-xylem transfer of sugars has also been described in several species, including woody species (Table 1 and references therein). Moreover, bidirectional movement of water and ions (e.g., potassium, sodium, phosphorus, calcium, and magnesium) in both angiosperms and gymnosperms (Table 1 and references therein) was reported. Lateral transport of solutes is thus an important component of resource allocation, as has been shown in several species [31] (Table 1 and references therein). However, most of these studies lacked specific data about the anatomical framework in which lateral transport takes place. Over the past 15 years, studies performed on the model plant *Arabidopsis thaliana* have given us a better picture of the cell types involved and have highlighted the role of vascular and perivascular cells in this process.

## 3. Going In and Out of the Vascular System: The Role of Specialized Vascular and Perivascular Cells

**Figure 1:**
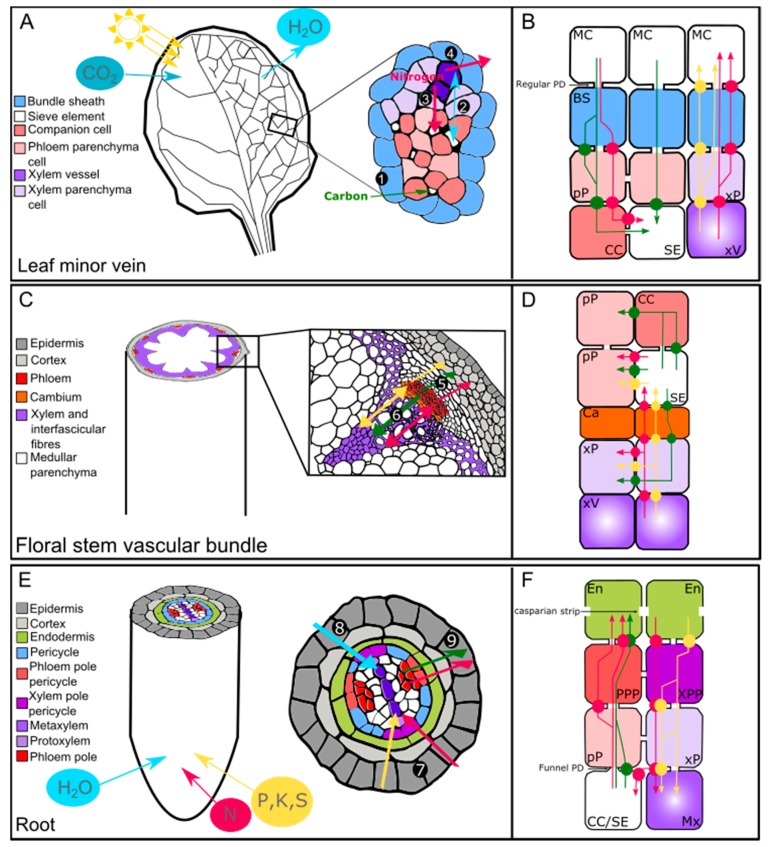
Organic and inorganic solutes take several paths to enter and exit the plant vascular system. This scheme is based on the *Arabidopsis thaliana* anatomy. (**A**,**C**,**E**) Schematic representation of a source organ (source leaf) (**A**), a transport organ (floral stem) (**C**), and a sink organ (root) (**E**). (**B**,**D**,**F**) Schematic representation of the possible transport pathways taken by the organic and inorganic solutes between the different cell types in each organ ((**B**): leaf; (**D**): floral stem; and (**F**): root). 1. Loading of carbohydrates, organic acids, and amino acids in the sieve tubes. 2. Water flow between xylem and phloem. 3. Lateral transfer of amino acids from xylem to phloem. 4. N metabolism and N remobilization. 5. Leakage and retrieval of carbohydrates, amino acids, and ions between the phloem and the surroundings tissues. 6. Unloading of carbohydrates, organic acids, amino acids, and ions for the supply of metabolic precursors for cell division and expansion. 7. Uptake, efflux, and influx of inorganic solutes (e.g., NO_3_^−^, PO_4_^3−^, K^+^, SO_4_^2−^) and nitrogen to the xylem. 8. Absorption and flow of water to the xylem. 9. Unloading of carbohydrates, organic acids, and amino acids that will later be used as precursors for cell division and expansion. The light blue, pale yellow, pink, and green arrows represent the water, inorganic solutes, nitrogen, and sugar movement, respectively. The circles represent the transporter-mediated movement of organic and inorganic solutes. BS: bundle sheath; CC: companion cell; En: endodermis; PD: plasmodesmata; Ph: phloem; pP: phloem parenchyma cell; PPP: phloem–pole pericycle; Px: protoxylem; MC: mesophyll cell; Mx: metaxylem; SE: sieve element; xV: xylem vessel; xP: xylem parenchyma cell; XPP: xylem-pole pericycle.

In higher plants, the vascular system is composed of phloem and xylem tissues, which are organized in an organ-specific manner (Figure 1). Anatomically, the phloem tissue is composed of phloem parenchyma cells, companion cells, and sieve elements, and mainly accounts for the long-distance transport of sugars [49], amino acids [50], phytohormones [51], and nucleic acids [52] from source (carbon fixing) to sink (carbohydrate import dependent) organs. The xylem tissue is composed of xylem parenchyma cells, xylem fibers, and xylem vessels that provide structural support, as well as enable the transport of water [53], minerals [1], and phytohormones [51] from below- to above-ground organs. Additionally, layers of specialized parenchyma cells and perivascular cells surrounding the phloem and xylem tissues, such as the pericycle in roots or the bundle sheath in leaves, also constitute an important interface involved in the lateral distribution of solutes between vascular cell types, as well as from vascular tissue to the outer cell layers, and vice versa.

### 3.1. The Bundle Sheath and Transfer Cells in Leaves

In leaves, phloem and xylem tissues face the adaxial and the abaxial part of the lamina, respectively, and are organized into bundles encircled by a parenchymatous layer of cells known as the bundle sheath (BS) [54] (Figure 1A). In previous studies, the BS cells of C_4_ plants have received the most attention because of their specialized role in efficient CO_2_ fixation in the Calvin–Benson cycle [55], while the role of BS cells in C_3_ plant leaves has been much less widely explored. Nevertheless, several studies performed on Arabidopsis indicate an important role for BS cells in the lateral transport of organic and inorganic solutes and suggest that BS cells facing phloem and xylem may play distinct roles [56]. In particular, those that face the xylem may form a selective barrier, preventing excessive amounts of ions entering or leaving the xylem [57]. In addition, they might also act as a hydraulic regulatory barrier to reduce loss of water from the veins during abiotic stress conditions [58]. In minor veins of source leaves, structural evidence suggests that sugars will cross the BS cells (those facing the phloem tissue), preferably through plasmodesmata, to reach the phloem parenchyma cells before being loaded into the phloem sieve elements [59] (Figure 1A,B). In addition, an apoplasmic route is also likely for the transport of organic and inorganic solutes between BS cells and vascular tissues, as suggested by the identification of candidate plasma membrane transporters detailed below [56,60,61,62] (Figure 1B).

Besides the important role of the bundle sheath in the lateral transport of solutes, other vascular cell types are involved in cell-to-cell transport between the phloem and the xylem and within each of these tissues. For instance, transfer cells (TCs), which can differentiate from existing vascular or non-vascular cell types (e.g., bundle sheath, xylem or phloem parenchyma cells), and which are characterized by prominent plasma membrane ingrowths, have been suggested to be a site of intense transport activity between the different vascular cell types [63]. Most studied in the phloem of minor veins, TC development occurs quite early (after an average of 10 days of growth) in cotyledons as well as in young leaves of *Arabidopsis thaliana* plants grown in normal conditions [64]. In adult leaves, a basipetal gradient of wall ingrowth deposition that has parallels with the sink/source transition is observed [64]. Additionally, TCs can develop in response to changing environmental conditions, most probably as a result of an increased demand for solute transport [65]. It has been proposed, for example, that phloem parenchyma transfer cells (PP TCs), which are highly metabolically active, could fuel massive transmembrane transport, notably of sugar compounds, through the plasmodesmata as well as through active and/or passive transporter systems [66]. Finally, the role of PP TCs in controlling membrane transport capacity has been extended to their role as a physical barrier that prevents access of invading pathogens to sugar-rich sieve elements [67]. Even if progress has been made in our understanding of the role of TCs, the molecular actors involved in the transport of organic solutes (i.e., sugar and amino acids) between TCs and phloem companion cells still need to be identified. On the basis of the localization of members of the SUGAR WILL EVENTUALLY BE EXPORTED TRANSPORTERS (SWEET) family in the plasma membrane ingrowths of TCs [68,69], the existence of a facilitated transport of sugars should be further explored.

### 3.2. Vascular Parenchyma Cells, Fibers, and Rays in the Stem

In *Arabidopsis thaliana*, the floral stem (also referred to as the inflorescence stem) is composed of collateral vascular bundles of phloem, cambium, and xylem, separated from each other by interfascicular fibers (Figure 1C). In particular, fibers referred to as bast fibers or xylary fibers (depending on whether they belong to phloem or xylem tissue) are found in the phloem and xylem [70]. Fibers can also be found in the hypocotyl phloem and xylem during secondary growth [71]. Even if the two types of fibers (i.e., xylary fibers and interfascicular fibers) do not originate from the same vascular cambium (i.e., the interfascicular cambium for interfascicular fibers and the fascicular cambium for xylary fibers), both provide structural support for the plant, thanks to their thick secondary cell walls [72]. It has recently been proposed that Arabidopsis xylary fibers also provide some of the monolignols used for xylem vessel lignification [15]. Xylem parenchyma cells (also referred to as xylary parenchyma cells) are also involved in supplying monolignols to the developing xylem vessels [15]. However, it is not currently known exactly which transport pathway is involved in this type of process. Certain members of the ATP-binding cassette (ABC) transporter family that have been shown to transport monolignols could be good candidates [73,74]. Additionally, whether or not exchanges between parenchyma cells/xylary fibers and developing vascular conducting cells exist for compounds other than monolignols (e.g., sugars) or occur in other tissues (e.g., phloem) remains an open question. Furthermore, if sugar exchanges do exist, questions of whether these are mediated through plasmodesmata, or active and/or passive transport pathways, represent interesting issues that should be addressed (Figure 1D), especially in terms of xylem secondary cell wall formation, which requires a significant amount of sugar to sustain cell wall polysaccharide synthesis.

Like in the Arabidopsis floral stem, in ligneous species, the woody stem is composed of xylem vessels and xylary fibers, but it also contains an additional cell type known as a ray. Rays, which are rarely found in the *Arabidopsis* floral stem and hypocotyl [71,75], are living cells responsible for the lateral transport of nutrients across the wood and for storage of carbohydrates [72]. Given their role, ray cells might thus be compared to the xylary parenchyma cells found in the Arabidopsis floral stem. In particular, it has been shown that during wood xylem development, there is an increasing requirement of sugars (i.e., glucose, fructose, and phosphate-sugars) that can be used for sustaining secondary cell wall formation [76]. Regarding the route taken by these sugars, they are most probably unloaded from the sieve elements into the wood rays and then reach the developing xylem vessels through a combination of symplasmic and apoplasmic pathways [77,78].

### 3.3. The Vascular Parenchyma Cells, Pericycle, and Endodermis in the Root

In a sink organ such as the Arabidopsis root, the young stele (composed of phloem, xylem, and vascular parenchyma cells, also known as stelar parenchyma) is surrounded by the pericycle and the endodermis (Figure 1E) [79]. As the roots get older, the pericycle cells start to divide and contribute, along with the dividing procambium, to the generation of the vascular cambium [80].

The term “stelar parenchyma” encompasses both the phloem parenchyma cells and the xylem parenchyma cells. In general, little attention has been given to the exact role of the root phloem parenchyma cells, while the root xylem parenchyma cells have received the most attention, mainly because of their role in ion/metal transport through the root. Nevertheless, experimental proof of lateral transport of organic and/or inorganic solutes between the phloem and the xylem through the vascular parenchyma cells in the roots is still lacking. Interestingly, a pioneering study of onion root (*Allium cepa*), which established a map of plasmodesmata distribution between the different vascular root cell types, identified several plasmodesmata at the interface between phloem–stelar and parenchyma–xylem vessels, suggesting a possible “direct” symplasmic pathway (i.e., without passing through the pericycle) for lateral transport (Figure 1F) [81].

The study conducted by Ma et al. (2001) also highlighted the fact that the pericycle would also play a significant role in the symplasmic transport of ions and sugars, both along the radial path and in a tangential direction across the radial walls (Figure 1F) [81]. However, direct proof of plasmodesmata-mediated transport of ions still needs to be provided. Later, a more refined analysis of the young root in the model plant Arabidopsis showed the existence of two types of pericycle cells; namely xylem-pole pericycle cells and phloem-pole pericycle cells (Figure 1E,F) [82]. Xylem-pole pericycle cells have been shown to be involved in lateral root formation as well as in the apoplasmic xylem loading of boron [83,84], while phloem-pole pericycle cells are specifically involved in the unloading of molecules from the phloem sieve elements [85]. In a recent study, Ross-Elliott et al. (2018), using a combination of imaging and mathematical modelling, accurately proved the existence of a novel type of plasmodesmata named funnel plasmodesmata, which are specifically responsible for connecting the phloem-pole pericycle cells to the protophloem sieve elements at the Arabidopsis root tip (Figure 1F). Ross-Elliott et al. suggest that sugars unloaded from the protophloem sieve elements move through the funnel plasmodesmata and are then able to access all other cell types through the “regular” plasmodesmata system (Figure 1F). Other molecules, such as macromolecules, are also able to pass through the funnel plasmodesmata, but are subsequently retained in the phloem-pole pericycle cells [85]. These results represent an important advance in our understanding of how these different cell types are connected, but also raise the question of whether funnel plasmodesmata exist in sink organs other than roots, and thus represent a potential sink-specific mode for sugar unloading.

The role of the endodermis as a bidirectional barrier that controls the solutes’ access to the vascular cylinder, as well as prevents nutrients from leaking out, is also quite well established [86,87]. Recently, it has been shown that, as in the pericycle, two endodermal cell types co-exist in roots and each cell type responds differently to nutrients and hormones [88]. It is also suggested that these cells display different uptake and sensing potential [88]. These findings add an extra level of complexity to the role of the endodermis in the lateral transport of solutes and raise the long-standing question regarding the coordination of lateral transport of solutes between the endodermis and the pericycle cells.

## 4. Molecular Actors Involved in Lateral Transport of Solutes

In the context of lateral transport, determining whether a solute follows an active or a facilitated transport pathway depends mostly on its relative concentration in the different tissue layers that it crosses. Thus, if a solute (e.g., sucrose) is more concentrated in the phloem sap than it is in the xylem sap, it is logical to assume that its phloem-to-xylem transfer will follow the concentration gradient through plasmodesmata and/or the action of facilitators. Conversely, xylem-to-phloem transfer of the solute would need an active transport step in order to overcome the concentration gradient.

Sugar gradients have been shown at the tissue level in *Vicia faba* cotyledons; in the *Ricinus communis* hypocotyl; in the *Populus tremula* wood forming region [76,89,90]; and, very recently, at the microscopic level in different Barley (*Hordeum vulgare*) and Arabidopsis organs [91]. In contrast, almost no direct experimental proof of inorganic gradients exists [3,92,93]. Nonetheless, over the last 15 years, numerous plasma membrane solute transporters that could be involved in the intercellular transport of solutes have been characterized. These are expressed either in the leaf, in the root, or in the stem vascular and perivascular cells of dicotyledonous species (Table 2). Interestingly, both active and passive transporters (i.e., facilitators) acting mostly at the interface between vascular parenchyma cells and conducting cells have been identified (Table 2).

### 4.1. Lateral Transport of Inorganic Solutes from Xylem-To-Phloem

Inorganic solutes can be divided into anions (i.e., nitrite, nitrate, chloride, sulfate, and phosphate) and cations (i.e., ammonium, potassium, sodium, calcium, and magnesium). The lateral transport of inorganic nitrogen (i.e., nitrite, nitrate, and ammonium) has been the most studied (for review, [23]), and some of the molecular actors have been characterized (Table 2). Other inorganic ions are also most probably transported from xylem to phloem through an apoplasmic and/or coupled trans-cellular pathway. Nonetheless, movement of inorganic ions through plasmodesmata, for example, between the pericycle and protoxylem in a sink organ such as the onion root (*Allium cepa*) [81], cannot be excluded. In this section, we will more specifically address transporters expressed in vascular and perivascular cells that could be good candidates for the lateral transport of potassium, sodium, and phosphate, because a substantial cycling of these ions has been shown [16,133].

#### 4.1.1. Potassium and Sodium

**Figure 2:**
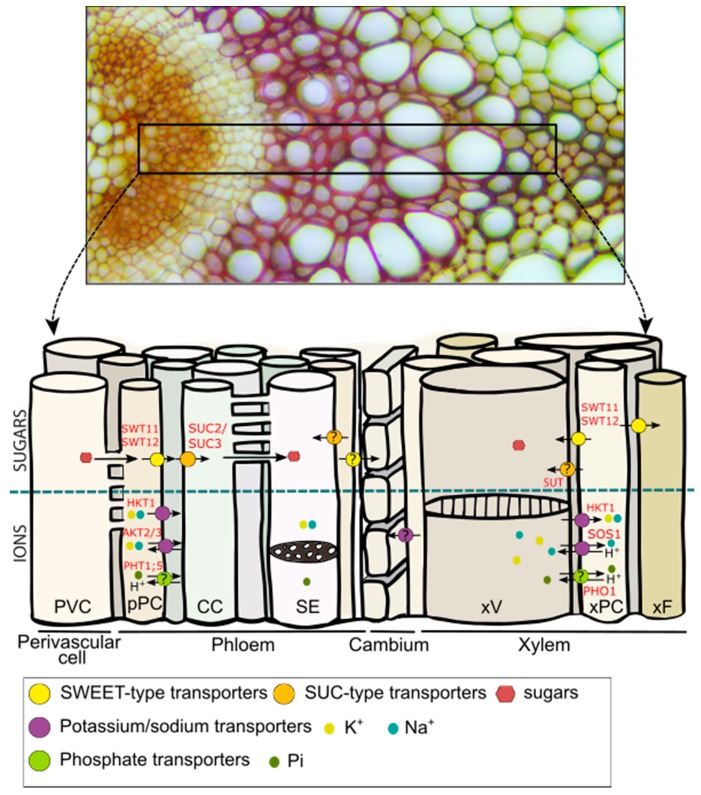
Model for sugar and ion transport in the Arabidopsis floral stem. The upper panel shows a vascular bundle transversal section stained with alcian blue/safranin O. The primary cell walls appear in different shades of orange and the secondary cell walls appear in red. The lower panel is a sketch showing a longitudinal view of the different cell types present in a vascular bundle. The white bridges between two cells represent the plasmodesmata. The model was based on current knowledge of the spatial distribution of sugar and ion transporters in the Arabidopsis floral stem. Known locations of members of the sugar and ion transporters in other Arabidopsis organ or in woody stem have been added as putative locations in Arabidopsis floral stem (question marks). pPC: phloem parenchyma cell; CC: companion cell; SE: sieve element; SUC: SUCROSE TRANSPORTER; SWEET: SUGAR WILL EVENTUALLY BE EXPORTED TRANSPORTERS; xV: xylem vessel; xF: xylary fiber; xPC: xylem parenchyma cell.

Potassium (K^+^) is the most abundant cation and is essential for many physiological processes [134]. Sodium (Na^+^), in contrast, has a dual role, being beneficial for plants at low concentrations, but becoming toxic as its concentration rises [134]. Sodium and potassium are often exchangeable, mainly because of their chemical similarities. The lateral transfer of Na and K, along with their long-distance transport, must thus be tightly coordinated to control the balance between the two. Both cations are taken up by the root system through numerous plasma membrane-localized transporters that are mostly expressed in the xylem parenchyma cells, such as the high-affinity potassium and sodium transporter 1 (HKT1), the cation/H^+^ exchanger (CHX14 and CHX21), the shaker-like outward channel (SKOR), and the Na^+^/H^+^ antiporter (SOS1) (Table 2 and references therein) [135]. Once loaded into the xylem sap through an active and/or a passive transport system [136], potassium and sodium are transported through the stem and unloaded at the target organs (e.g., the leaf) through the action of the high affinity potassium/sodium symporter (HKT1) (Table 2 and Figure 2). In addition, phloem/xylem sap analysis using radioactive isotopes of potassium and sodium showed that both cations are also partially transferred from the xylem to the phloem to be sent back to the root [25,31,137]. This process has been studied in particular in *Ricinus communis* [31]. In this species, more than 30% of the total Na^+^ taken up in the root is actually recycled back to the root after a xylem-to-phloem transfer. Additionally, half of the K^+^ loaded into the xylem sap is recycled back via the phloem to be incorporated into the roots. Even if this general flow model emphasizes the role of the lateral transfer between xylem and phloem for both cations in this species [31], the question of whether or not the unloading of potassium and sodium from the root phloem occurs through plasmodesmata needs to be further addressed.

Peuke [31] also noted that, in terms of proportion, the general flow model for K^+^ is quite similar to that of the N, suggesting a strong interaction between the two compounds. In tomato, the K^+^ concentration in the xylem sap has been shown to influence the rate of amino acid uptake, with a low K^+^ concentration stimulating amino acid uptake, and vice versa (Table 2 and references therein). More broadly, it has been shown that each time a substrate is taken up through a proton symport, K^+^ ions are released into the transpiration stream [138]. Regarding the translocation stream, analysis of mutants deficient in the expression of the AKT2/3 potassium channel localized in the phloem parenchyma cells in the Arabidopsis floral stem (Figure 2) showed that the retrieval mechanisms of sugars along the phloem tissue are also intimately linked to the transport of K^+^ ions via the action of AKT2/3 [139]. Subsequent studies identified that K^+^ ions moving in the phloem sap can be assimilated into an energy store that can be used to overcome local energy limitations generated by the action of the K^+^ and sugar proton-coupled transporters [140,141].

As AKT2/3 is a channel that is responsible for the facilitated diffusion of potassium, its mode of action implies the presence of a potassium gradient between the phloem companion cell/sieve element complex and the surrounding tissues that can be used locally to assure the retrieval and subsequent reloading of sugar by and from the transport phloem [141]. However, the exact nature of the transporters involved in such a process is still not known with certainty.

Sodium ions can also be loaded into the root xylem vessel via SOS1, a xylem parenchyma-localized antiporter, which mediates Na^+^/H^+^ exchanges [108]. Interestingly, SOS1 is also located in the xylem parenchyma cells of the floral stem and leaves [108], suggesting that this transporter could work both to load sodium into the xylem vessel and to retrieve it along the transpiration stream (Figure 2). Once in the transpiration stream, it has been suggested that sodium could be laterally transferred from xylem to phloem, likely via the action of the HKT1 transporter. This transfer has been suggested to be biologically relevant in order to prevent ion overaccumulation in the shoot of Arabidopsis or cotton in the context of high salinity [142,143]. However, Davenport et al. [144] later challenged the role of HKT1 in this process and, therefore, the very existence of sodium recirculation to the root through the phloem has been questioned and remains under debate [145]. One way to address this question would be to estimate the relative Na^+^ fluxes in the phloem and xylem sap. While in *Ricinus communis*, the recirculation of sodium has been estimated to be around 30%, similar experiments in monocotyledons suggest that lateral transfer of sodium would only account for 5–7% of the total sodium uptake [145]. Without questioning the existence of sodium recirculation, these discrepancies point out the need to carefully estimate phloem and xylem flow rates according to the plant species and environmental conditions.

#### 4.1.2. Phosphate

Phosphorus (P) is a macronutrient essential for cellular processes such as energy production, redox reactions, photosynthesis, and phosphorylation/dephosphorylation reactions [146]. Phosphorus enters the plant root system in the form of inorganic P (Pi), such as PO_4_^3−^, H_2_PO_4_^−^, or HPO_4_^2−^, through an energized process involving H^+^/Pi co-transport in order to overcome the negative membrane potential [146]. Pi is then transported through the different root tissues through the action of, at least, the AtPHO1 and AtPHO1;H1 transporters, which are expressed in the endodermis and the pericycle, respectively, before being loaded into the xylem sap for root-to-shoot transport [102,103]. Additionally, under long-term Pi starvation, Pi is remobilized from the old leaves toward the sink tissues (e.g., the growing root or the seeds). This remobilization from shoot-to-root requires the action of the phloem-localized PHT1;5 transporter [104,147]. Although phosphorus was the first anion for which lateral transport was shown [25], it is still not known with certainty which transporters are involved in Pi recycling from the xylem to the phloem, although the antiporters PHO1 and PHT1;5 could be involved in such a process (Figure 2). In addition, it has been suggested that in rice, Pi might also be transported by members of the SULTR transporter family [148]. As most phosphate transporters are conserved between monocots and dicots, assessing the role of SULTR transporters in the lateral transfer of Pi may also be relevant. To improve our limited knowledge of lateral transport of Pi, one of the first steps will thus be to carefully identify the vascular cell types in which the plasmalemmal Pi transporters are expressed. Once this is achieved, genetic tools and Pi flow modelling could then be combined to help to better define the contribution of long-distance versus lateral Pi transport.

### 4.2. Lateral Transport of Sugars in the Vascular System

Experimental proof for the lateral transport of sugars has been provided for both the phloem-to-xylem and the xylem-to-phloem direction (Table 1). This transport is thought to primarily act in carbon transport and recirculatization via the phloem or the xylem. At a more local scale, the leakage-retrieval mechanism of sugars (recruiting both plasmodesmata or facilitators for leakage and energized transporters for retrieval) that occurs along the transport phloem (for review, [17]) can also be regarded as the lateral transport process.

#### 4.2.1. Leakage-Retrieval Process along the Transport Phloem

The first evidence for a leakage-retrieval process along the transport phloem was obtained in experiments performed on the *Phaseolus vulgaris* stem [42,149,150,151,152]. This was demonstrated by the combined use of ^11^C-labelled substrate, electron microscopy techniques, and application of *p*-chloromercuribenzene sulphonic acid (PCMBS), which blocks active sugar loading into the phloem [42,149,150,151,152]. In *Phaseolus vulgaris*, passive leakage of photosynthates from the phloem sieve elements was shown to occur at a rate of 4% cm^−1^. The lost sugars were then assumed to be continuously reloaded by active transport [152]. In Arabidopsis, the sucrose/H^+^ symporter SUC2, located at the plasma membrane of the companion cells, has been shown to play a role in retrieving leaked sucrose along the transport phloem in addition to having a well-known role in loading sucrose into the phloem sieve elements [45] (Figure 2). Another part of the leaked sucrose is used by the lateral sinks, such as the cambium and possibly the xylem, for growth and tissue maintenance [17]. However, the exact proportion of sucrose involved in this leakage-retrieval cycle, as well as the molecular actors involved, remain difficult to determine. Nevertheless, observation of a partial complementation of the *suc2* mutant line by expression of the *SUC2* gene under control of the minor vein-specific galactinol synthase promoter from melon (*CmGAS1*) in this mutant suggests that the retrieval mechanism is not negligeable [153]. It is worth mentioning that this type of leakage-retrieval process most likely depends on the plant species, developmental stage, and growth conditions [154].

#### 4.2.2. Phloem-To-Xylem and Xylem-To-Phloem Transport of Sugars in the Stem

In tree species, a proposed important role of the lateral transport of sugars is the prevention and/or repair of xylem embolism and/or cavitation. Transport of sugars between vascular cell types has been demonstrated during the xylem refilling process that occurs following an embolism [155]. It has been hypothesized that, during such a phenomenon, movement of water and sugars stored in the phloem towards the xylem vessels could be used to repair the embolism [155]. Additionally, as suggested for the walnut tree (*Juglans regia*), cell-to-cell transport of sugars between ray cells and xylem vessels, most likely mediated by a SUC2/SUT1 ortholog, could also account for the embolism repair [113] (Figure 2). Furthermore, it has been hypothetized that the starch stored in ray cell xylem parenchyma cells would depolymerize during xylem refilling and that the sugars produced during this phase are then loaded into cavitated conduits. In turn, the reduced level of starch in the xylem parenchyma cells would result in the cells becoming strong sinks, with the consequent unloading of sugars and water from the phloem directed to the refilling conduits [155]. The difference in sugar concentration between the two compartments/tissues would imply preferential use of plasmodesmata or a facilitated sugar transport system to sustain such a movement. Here, the sugar efflux transporters of the SWEET family could be good candidates [156].

In the Arabidopsis floral stem, the formation of xylem fibers and vessels and the subsequent extra-thickening of their secondary cell walls also appears to rely on lateral movement of sugars [121]. The existence of a sugar gradient between xylary parenchyma cells and xylem vessels has been suggested in a study of the double mutant *sweet11sweet12* [121]. *SWEET11* and *SWEET12* are encoded for facilitators that transport sugars along the concentration gradient without any energized process [68], and are expressed in the phloem and xylem parenchyma cells of the floral stem [121] (Figure 2). The *sweet11sweet12* double mutant was found to exhibit defects during development of the floral stem vascular system, as well as a modified xylem secondary cell wall composition [121]. Modification of the phloem cell wall composition has also been shown in the double mutant line using synchrotron-based Fourier-transformed infrared spectroscopy (FTIR) [157], suggesting that facilitated sugar transport between vascular parenchyma cells and developing conducting cells (i.e., xylem vessels, xylary fibers, and phloem sieve elements) is required to sustain normal cell wall formation. However, whether this movement takes place in the phloem-to-xylem direction or more locally between vascular parenchyma cells and conducting cells needs to be further addressed.

Moreover, it is not yet known if a similar transport mode exists in other plant species, such as ligneous species, in particular during wood formation, which requires a high amount of sugars for the synthesis of cell wall polysaccharides and lignin. The recent release of the aspen transcriptome profiling datasets, with a resolution of a few microns in the wood forming region, has enabled analysis of the expression pattern of *SWEET* genes during xylem formation [78,158]. Analyses have shown that several *SWEET* genes are also expressed in this region, supporting the possible participation of PtSWEET11/12 in the sugar exchange between rays and xylem vessels [78]. Interestingly, in aspen, it has been shown that once in the apoplasm of xylem tissue, sucrose can be delivered to the developing vessels through the action of the PttSUT3 (sucrose/H^+^ symporter) to support the secondary cell wall formation [77]. In addition, increased levels of hexoses and hexose phosphates, as well as UDP–glucose, in cells undergoing secondary cell wall formation have been identified across the wood forming region in poplar. This confirms the higher need for sugars in these cells and the existence of a sugar gradient along the xylem area undergoing secondary cell wall maturation and cell death [76]. A similar role for the SUT1 transporter has also been hypothesized in *Solanaceous* species (tomato, potato, and tobacco) because of its presence in both the phloem and the xylem parenchyma cells [119]. Further studies of members of the SUC/SUT and SWEET family will be needed to refine their possible role in the lateral transport of sugars (Figure 2).

#### 4.2.3. Lateral Transport of Sugar in the Leaf

Even if there is no evidence for lateral transport of sugars between the vascular tissues in leaves, previous studies have identified interesting candidates that could be involved in the cell-to-cell transport of organic solutes between leaf bundle sheath and vascular tissues. Cui et al. (2014), using ChIP-chip experiments, identified several targets of the SHORT-ROOT (SHR) and/or SCARECROW-LIKE23 (SCL23) transcription factors, which specify the fate of bundle sheath cells in Arabidopsis leaves. Among them, some are involved in sugar transport, including the hexose transporters *PMT5* and *STP1* and the sucrose transporter *SUC1*. Interestingly, additional targets identified in this study are known to be involved in nitrogen (*NFP6.3*, *NPF4.6, PROT3*, *UMAMIT1*, and *CAT6*), potassium (*KUP1* and *KT12*), magnesium (*MGT9/MRS2-2* and *MGT6/MRS2-4*), and phosphate transport (*PHT1;3*) [159]. Furthermore, by characterizing the SHORT-ROOT/SCARECROW/SCARECROW-LIKE23 (SHR/SCR/SCL23) regulatory module, Cui et al. (2014) showed that in leaf major veins, *SCR* is preferentially expressed in BS cells facing the phloem, while *SCL23* is expressed in BS cells facing the xylem. This suggests that, depending on their closest cell neighbors, BS cells could fulfil specific roles [159].

Additionally, cell-specific transcriptomic studies in Arabidopsis allowed the identification of differentially expressed genes in the leaf bundle sheath cells [61,62]. In particular, they identified genes coding for sugar transporters (i.e., *AtSWEET10*, *AtSUC8*), as well as amino acid/organic acid transporters (i.e., *AtGLR3.6*, *AtALMT6*).

Altogether, these works suggest the existence of a fine regulation of lateral solute transport mediated by an active and/or a passive transport system, and provide evidence that the lateral transport also integrates oriented flow of organic and inorganic solutes, either towards the adaxial or the abaxial side of the lamina.

## 5. State-Of-The-Art Biophysical Tools for Monitoring in Situ Lateral Transport of Solutes

A better understanding of lateral transport of solutes in vascular tissues requires correct description of the types of connections that exist between the vascular cells. This is particularly important for anatomically highly complex organs such as the stem. In the 1980s to 1990s, this issue was addressed using transmission electron microscopy, which allows plasmodesmata frequency maps between cells to be determined and, therefore, symplasmic continuity or discontinuity to be identified [160,161]. In addition, in rice leaf blades, combined use of fluorescent markers and confocal scanning laser microscopy has enabled identification of a symplasmic phloem-loading pathway between xylem parenchyma cells and phloem sieve elements [162]. 

In addition, Liesche et al. (2012) developed the three-dimensional photoactivation microscopy technique in order to quantify plasmodesmata-mediated cell wall permeability between different cell types with cage fluorescein as tracer, enabling them to quantify the real-time mobility between cells in angiosperms and gymnosperms [163,164]. As it is now possible to synthetize conjugated-molecules, such as fluorescent phytohormones [165,166], which are mobile between different cell types, and hydrophilic gold nanoparticles, which can be transported in the vascular tissues [167], the development of conjugated sugars might also be used as a more direct approach to trace sugars at the tissue or cell level. In line with these techniques, the fluorescent coumarine glucoside esculin, which mimics sucrose translocation [168], is a promising tool for exploring lateral transport. The use of infrared spectroscopy (FTIR) combined with state-of-the-art statistical modeling has also been proven effective for quantitatively imaging the sucrose content at the microscopic level in barley leaf, stem, and seeds, as well as in Arabidopsis hypocotyls [91]. This breakthrough technology thus represents a great opportunity to better characterize the lateral transport of metabolites in the future.

Lateral exchanges of sugars and amino acids might also be monitored at a higher spatial resolution using isotope tracers such as ^15^N and ^13^C/^11^C and cryo-secondary ion mass spectroscopy. This technique has already been successfully used to demonstrate lateral exchanges of magnesium, potassium, and calcium between vascular tissues in the french bean (*Phaseolus vulgaris*) stem [3]. This type of mineral ion movement has also been observed in planta with the real-time radioisotope imaging system (RRIS) [169]. This technique also allowed the authors to follow the temporal evolution of ^14^C-labelled metabolites over 24 h in the different organs at the whole plant level [169]. Finally, the use of new imaging techniques in plant sciences, such as position emission tomography (PET), which exploits positron-emitting radioactive metabolite analogues, will provide exciting opportunities to improve our understanding of solute flows within the vascular system [170]. State-of-the-art techniques such as these will likely be required to better understand the lateral movement of a wide range of compounds including sugars and inorganic ions.

## 6. Concluding Remarks and Future Prospects

Since the existence of lateral movement of inorganic solutes was first reported in the 1930s, significant progress has been made in identifying several of the molecular actors involved in organic and inorganic solute exchanges. It has been discovered that the lateral transport of sugars is controlled by coordinated symplasmic and apoplasmic pathways, which constitutes a new paradigm that still needs to be confirmed for the lateral transfer of other solutes. In this scheme, another important step will be to better understand the environmental factors regulating the relative contribution of both pathways, as well as to take into account that their contributions probably vary between organs within a plant species and between plant species. Thus, more knowledge needs to be acquired at the cell level (e.g., plasmodesmata density, localization of the solute transporters) before the relative contributions of both pathways to the lateral transport can be determined. In addition, the use of cell-specific and single-cell technology combined with next generation sequencing would help to identify additional transporter families that could be involved in lateral movement [60,171]. For instance, the use of bundle sheath (e.g., *SHR* and *SCR23*), xylem parenchyma (e.g., *PRX47*), or phloem parenchyma (e.g., *GIGANTEA*) specific promoters combined with the translatome tibosome affinity purification (TRAP) technology, developed by Mustroph et al. [60], could help to provide a better understanding of the specificity of these cell types, as well as to go further in the identification of transporters involved in solute loading and unloading between vascular and perivascular cells.

Overall, addressing these questions will help to better comprehend how the carbon, ion, and nitrogen allocation and the interaction between them are coordinated throughout the plant body. This knowledge could finally be used to achieve a complete model of the resource economy in plants and thus to improve crop yield.

## Figures and Tables

**Table 1:** Experimental evidence for lateral transport of solutes in angiosperms and gymnosperms.

Lateral Process	Compound	Method Used	Species	Reference
Xylem-to-phloem	Sucrose	^14^C-sucrose	*Populus deltoids*	[32]
	Glumatic acid, aspartic acid	^14^C-labeled amino acid	*Populus deltoids*	[32]
	Valine, asparagine, threonine, serine, citrulline, glutamine	^14^C-labeled amino acid	*Lupinus albus*	[33]
	Asparagine	^14^C-asparagine	*Spartium junceum*	[34]
	Glucose	^14^C-glucose	*Vitis vinifera*	[35]
	Inulin, valine, aminobutyric acid	^14^C-labeled compounds	*Lycopersicum esculentum*	[28]
	Carbon and nitrogen	C/N ratio, sap analysis, and modeling	*Glycine max*	[30]
	Potassium and sodium	Sap analysis	*Salix viminalis*	[26]
	Potassium, sodium, and phosphorus	Radioactive isotopes	*Pelargonium zonale* *Salix lasiandra*	[25]
	Sucrose, glucose, and fructose	^14^C-labeled sugars	*Salix viminalis*	[36]
	Glutamic acid, aspartic acid	^14^C-labeled amino acid	*Salix viminalis*	[37]
Phloem-to-xylem	Photosynthates	Physiological analysis	*Ricinus communis*	[31]
	Photosynthates	^14^CO_2_	*Vitis labruscana*	[38]
	Glucose, fructose, and sucrose	^14^C-labeled sugars	*Glycine max*	[39]
	Sucrose	^14^CO_2_	*Salix viminalis*	[40]
	Phosphorus and sulfur	^35^S and ^32^P tracking experiments	*Phaseolus vulgaris*	[41]
Leakage-retrieval process along the phloem	Photosynthates	^11^CO_2_	*Phaseolus vulgaris*	[42]
	Sucrose and hexoses	Ringing experiment	*Gossypium* sp.	[43]
	Photosynthates	Defoliation experiment	*Fraxinus americana*	[44]
	Sucrose	^11^CO_2_ tracking experiment	*Arabidopsis thaliana*	[45]
Bidirectional exchange	Glutamine and asparagine	^14^C- and ^15^N-labeled compounds	*Picea abies*	[46]
	Potassium and sodium	Sap analysis	*Lupinus albus*	[47]
	Potassium	Sap analysis and modeling	*Ricinus communis*	[31]
	Potassium	Sap analysis	*Hordeum vulgare*	[47]
	Calcium and magnesium	Sap analysis	*Ricinus communis*	[48]
	Water	Fluorescent marker	*Eucalyptus saligna*	[5]

**Table 2:** Plasma membrane solute transporters showing a localization in vascular or perivascular cells in dicotyledonous species.

Gene Name	Gene Locus or Accession n°	Function	Organ	Expression Domain	Method Used	Species/[Reference]
**Ion transport**						
AKT2/3	At4g22200	Potassium/sodium channel	Leaf, stem	Phloem and xylem parenchyma	Promoter GUS fusion	At/[94,95]
CCC	At1g30450	Cation–chloride cotransporter	Leaf, root	Xylem parenchyma	Promoter GUS fusion	At/[96]
CHX14	At1g06970	Potassium efflux transporter	Root	Xylem parenchyma	Promoter GUS fusion	At/[97]
CHX21	At2g31910	Sodium transporter	Root	Endodermis	Protein immunolocalization	At/[98]
HKT1	At4g10310	Sodium/Potassium symporter	Leaf, root and stem	Phloem/xylem parenchyma, endodermis	Immuno-electron microscopy and promoter GUS fusion	At/[99,100]
NRT1.5/NPF7.3	At1g32450	Potassium and nitrate transporter	Root	Pericycle	In situ hybridization	At/[101]
PHO1	At3g23430	Inorganic phosphate exporter	Root	Stele, xylem-pole, endodermis	Promoter GUS fusion	At/[102]
PHO1;H1	At1g68740	Inorganic phosphate transporter	Leaf, root	Vascular cylinder, pericycle	Promoter GUS fusion	At/[103]
PHT1;5	At2g32830	Inorganic phosphate transporter	Leaf	Phloem	Promoter GUS fusion	At/[104]
GmPT7	FJ814695	Phosphate transporter	Leaf, root	Vascular cylinder	Promoter GUS fusion	Gm/[105]
SKOR	At3g02850	Potassium efflux transporter	Root	Xylem parenchyma, pericycle	Promoter GUS fusion	At/[106]
SLAH1-3	At1g62280; At4g27970; At5g24030	Anion channel	Root	Xylem-pole pericycle	Promoter GUS and GFP fusion	At/[107]
SOS1	At2g01980	Sodium antiporter	Leaf, stem, root	Xylem parenchyma, pericycle	Promoter GUS fusion	At/[108]
SULTR2;1	At5g10180	Sulfate transporter	Leaf, root	Xylem parenchyma, phloem, pericycle	Promoter GUS and GFP fusion	At/[109]
SULTR2;2	At1g77990	Sulfate transporter	Leaf, root	Bundle sheath, phloem parenchyma	Promoter GUS and GFP fusion	At/[109]
SULTR3;5	At5g19600	Sulfate transporter	Root	Xylem parenchyma, pericycle	Promoter GFP fusion	At/[110]
PtaSULTR1;1	DQ906929	Sulfate transporter	Stem	Phloem companion cells, cambium	In situ hybridization	Pta/[111]
PtaSULTR3;3a	DQ906924	Sulfate transporter	Leaf, stem	Companion cells, xylem parenchyma, rays	In situ hybridization	Pta/[111]
**Sugar transport**						
DcSUT2	Y16768	Sucrose transporter	Root	Xylem and phloem parenchyma	Northern blot	Dc/[112]
JrSUT1	AY504969	Sucrose transporter	Stem	Xylem parenchyma	In situ hybridization	Jr/[113]
PtaSUT3	POPTR_0019s11560	Sucrose transporter	Stem	Xylem vessel, fiber	In situ hybridization	Pta/[114]
PttSUT3	POPTR_0019s11560	Sucrose transporter	Stem	Cambium, xylem vessels	Gene expression by qPCR	Ptt/[77]
SUC2	At1g22710	Sucrose transporter	Leaf	Phloem companion cells	Promoter GUS/GFP fusion	At/[115,116]
SUC3	At2g02860	Sucrose transporter	Leaf, stem	Phloem companion cells	Protein immunolocalization and promoter GFP fusion	At/[117,118]
SUT1	X82276 (Nt); X82275 (Sly); X69165 (St)	Sucrose transporter	Leaf	Xylem parenchyma	Protein immunolocalization	St, Nt, Sly/[119]
SWEET4	At3g28007	Hexose facilitator	Root	Stele	Promoter GUS fusion	At/[120]
SWEET11	At3g48740	Sucrose and hexose facilitator	Leaf, stem	Phloem transfer cells, phloem/xylem parenchyma	GFP fusion protein	At/[68,121]
SWEET12	At5g23660	Sucrose and hexose facilitator	Leaf, stem	Phloem transfer cells, phloem/xylem parenchyma	GFP fusion protein	At/[68,121]
IbSWEET10		Sucrose transporter	Root	Stele	Promoter GUS fusion	Ib/[122]
**Organic and inorganic nitrogen**						
AAP2	At5g09220	Amino acid transporter	Leaf, stem	Phloem companion cells	Promoter GUS and GFP fusion protein	At/[18]
AAP6	At5g49630	Amino acid transporter	Leaf	Xylem parenchyma	Promoter GUS	At/[123]
AMT1;1	At4g13510	Ammonium transporter	Root	Pericycle	GFP fusion protein	At/[124]
AMT2;1	At2g38290	Ammonium transporter	Root	Pericycle	Promoter GFP	At/[125]
AtProT1	At2g39890	Proline transporter	Leaf, root	Phloem and phloem parenchyma	Promoter GFP	At/[126]
NPF2.3	At3g45680	Nitrate transporter	Root	Pericycle	Promoter GUS and GFP fusion protein	At/[127]
NRT1.4/NPF6.2	At2g26690	Nitrate transporter	Leaf	Vascular system	In situ hybridization	At/[128,129]
NRT1.8/NPF7.2	At4g21680	Nitrate transporter	Root	Xylem parenchyma	Promoter GUS and in situ hybridization	At/[130]
NRT1.9/NPF2.9	At1g18880	Nitrate transporter	Root	Phloem companion cells	Promoter GUS and GFP fusion protein	At/[131]
NRT1.11/NPF1.2	At1g52190	Nitrate transporter	Leaf	Phloem companion cells	Promoter GUS and GFP fusion protein	At/[132]
NRT1.12/NPF1.1	At3g16180	Nitrate transporter	Leaf	Phloem companion cells	Promoter GUS and GFP fusion protein	At/[132]

At: *Arabidopsis thaliana*; Bv: *Beta vulgaris*; Dc: *Daucus carota*; Gm: *Glycine max*; Ib: *Ipomoea batatas*; Jr: *Juglans regia*; Nt: *Nicotiana tabacum*; Vv: *Vitis vinifera*; Pta: *Populus tremula x Populus alba*; Ptt: *Populus tremula x Populus tremuloïdes*; Sly: *Solanum lycopersicum*; St: *Solanum tuberosum*.
